# Books and Pamphlets Received

**Published:** 1884-08-20

**Authors:** 


### BOOKS AND PAMPHLETS RECEIVED.

*The Sanitarian* for July, 1884.

A very interesting number of this valuable Journal.

*How to Grow Fine Celery.* A New Method, by Mrs. H. M. Crider, York, Pa. Price, twenty-five cents.

*An Ephemeris of Materia Medica, Pharmacy, Therapeutics and Collateral Information*, by Edward R. Squibb, M.D., Edward H. Squibb, M.D., Chas. F. Squibb, M.D., Brooklyn, N. Y., July, 1884.

This Journal is filled with its usual amount of interesting and practical information.

*The Tribute of Praise and Methodist Protestant Hymn Book*, edited by Dr. Eben Tourjee. The Board of Publication of the Methodist Protestant Church, Pittsburgh. Wm. McCracken, Jr., Baltimore. W. J. C. Dulany: 1883.

Among the many new and excellent Song and Hymn Books recently published, the above work holds, deservedly, a high place. The selections are excellent, and make up a large collection suited to the Church and Sabbath-school, and, also, to social meetings.

*The Medical Graduate and His Needs,* by George C. Wellner, M.D. Published by George S. Davis, Detroit, Michigan.

We recommend the above little book to the young "medico." To use the language of the author, its being the only one of the kind may serve as its raison d'etre. The customary challenge, "Why do ye spend money and time for that which is not bread?" does not apply to it.

Although designed chiefly for students and young practitioners, it can be read with profit by "children of a larger growth."

*Quarantine and Sanitary Operations of the Board of Health of the State of Louisiana* during 1880, 1881, 1882, 1883, by Joseph Jones, M.D., President of the Board of Health of the State of Louisiana. Introduction to the Annual Report of the Board of Health to the General Assembly of the State of Louisiana, 1883-'84.

This is an interesting and important work, containing useful statistical information connected with sanitary matters in Louisiana. It contains 390 oc. pages, and is gotten up with that ability and accuracy of detail for which the works of Prof. Jones are noted.

*Transactions of the Medical Society of the State of Tennessee,* 1884. Fifty-first Annual Meeting, held at Chattanooga, April, 1884.

The work is plainly, but neatly gotten up, and contains 139 oc. pages.

The address of welcome was made by Dr. H. L. McReynolds, of Chattanooga, in appropriate language.

The retiring President made an interesting address on the subject, "To Medical Men Belong Medical Matters."

The following papers were read before the Society, to-wit: Diphtheritic Sequelæ, Prof. W. M. Vertrees, M.D.; Eczema, R. F. Evans, M.D.; Efforts of Therapeutics, H. Berlin, M.D.; Excitation of the Clitoris, Chas. E. Ristine, M.D.; Hospital Gangrene, J. R. Rathmell, M.D; Hot Water Therapeutics, Mary T. Davis, M.D.; Hygiene of the Eye, J. M. Masters, M.D.; Melanotic Sarcoma (?), Prof. J. G. Sinclair, M.D.; Puerperal Fever, A. J. Swaney, M.D.; Scrofula, John Blankenship, M.D.; Senile Prostatic Disease, S. T. Hardison, M. D.; Small-pox in Chattanooga, 1882-'83, E. M. Eaton, M D.; State Board of Health.

Officers elect for ensuing year:

President—D. D. Saunders, M.D., Memphis.

Vice Presidents—For Middle Tennessee, A. J. Swaney, M.D, Castallain Springs; for West Tennessee, T. J. Reed, M.D., Mason; for East Tennessee, W. T. Hope, M.D., Chattanooga.

Secretary—C. C. Fite, M.D., Nashville.

Treasurer—Deering J. Roberts, M.D., Nashville.

The next meeting is appointed at Nashville, the second Tuesday in April, 1885.

